# Characterization and Specific Detection of *Lactobacillus paracasei*-Derived Extracellular Vesicles Using Anti-p40-Modified Au Thin Film

**DOI:** 10.3390/pharmaceutics17050654

**Published:** 2025-05-16

**Authors:** Kyeongmin Lee, Eun-Gyung Cho, Youngbo Choi, Yunsik Kim, Jin Hee Lee, Surin Hong

**Affiliations:** 1 Department of Biotechnology, CHA University, Pocheon 11160, Gyeonggi, Republic of Korea; kmlee04@naver.com; 2 Consumer Health 2 Center, CHA Biomedical Research Institute, CHA Bundang Medical Center, CHA University School of Medicine, Seongnam 13496, Gyeonggi, Republic of Korea; egcho@chamc.co.kr (E.-G.C.); spcp@chamc.co.kr (Y.K.); mapcar@chamc.co.kr (J.H.L.); 3 H&B Science Center, CHA Meditech Co., Ltd., Seongnam 13488, Gyeonggi, Republic of Korea; 4 Department of Life Science, General Graduate School, CHA University, Pocheon 11160, Gyeonggi, Republic of Korea; 5 Department of Safety Engineering, Chungbuk National University, Cheongju 28644, Chungbuk, Republic of Korea; ybc@chungbuk.ac.kr; 6 Department of BigData, Chungbuk National University, Cheongju 28644, Chungbuk, Republic of Korea

**Keywords:** extracellular vesicles, exosomes, *Lactobacillus paracasei*, characterization, surface plasmon resonance, quantification

## Abstract

**Background/Objectives**: Extracellular vesicles (EVs) are nanoscale, membrane-enclosed structures that play key roles in intercellular communication and biological regulation. Among them, *Lactobacillus paracasei*-derived EVs (Lp-EVs) have attracted attention for their anti-inflammatory and anti-aging properties, making them promising candidates for therapeutic and cosmetic use. However, methods for specific detection and quantitative evaluation of Lp-EVs are still limited. This study aims to develop a surface plasmon resonance (SPR)-based sensor system for the precise and selective detection of Lp-EVs. **Methods**: Anti-p40 antibodies were immobilized on gold thin films to construct an SPR sensing platform. The overexpression of the p40 protein on Lp-EVs was confirmed using flow cytometry and Western blotting. For functional evaluation, Lp-EVs were applied to an artificial skin membrane mounted on a Franz diffusion cell, followed by SPR-based quantification and fluorescence imaging to assess their skin penetration behavior. **Results**: The developed SPR sensor demonstrated high specificity and a detection limit of 0.12 µg/mL, with a linear response range from 0.1 to 0.375 µg/mL. It successfully discriminated Lp-EVs from other bacterial EVs. In the skin diffusion assay, Lp-EVs accumulated predominantly in the epidermal layer without penetrating into the dermis, likely due to their negative surface charge and interaction with the hydrophobic epidermal lipid matrix. Fluorescence imaging confirmed this epidermal confinement, which increased over 24 h. **Conclusions**: This study presents a sensitive and selective SPR-based platform for detecting Lp-EVs and demonstrates their potential for targeted epidermal delivery. These findings support the use of Lp-EVs in skin-focused therapeutic and cosmetic applications. Future studies will explore strategies such as microneedle-assisted delivery to enhance transdermal penetration and efficacy.

## 1. Introduction

Extracellular vesicles (EVs) are cell-derived membrane-surrounded vesicles, ranging in size from 50 to 200 nm in diameter, that play a crucial role in intercellular communication. Composed of a lipid bilayer, EVs encapsulate various biomolecules, including proteins, lipids, DNA, and RNA, making them essential in biological functions such as serving as biomarkers, drug carriers, and therapeutic agents [1,2]. For instance, levels of EVs vary depending on disease states, and these fluctuations can be utilized as biomarkers for the diagnosis of diseases like cancer and tumors [3,4,5,6]. Moreover, due to their high biocompatibility, EVs are effective vehicles for drug delivery. They have been employed to deliver therapeutic agents for the treatment of conditions such as colorectal cancer, breast cancer, tumors, and inflammation [7,8,9,10]. Additionally, certain exosomes, such as those derived from mesenchymal stem cells (MSCs), are reported to possess intrinsic therapeutic properties, including skin regeneration and immune modulation [11,12,13,14]. Given their potential as disease biomarkers, drug delivery systems, and therapeutic agents, it is crucial to develop analytical techniques capable of accurately quantifying exosome concentrations for clinical applications.

One of the techniques commonly employed for the analysis of EVs is the Enzyme-Linked Immunosorbent Assay (ELISA). This method leverages antigen–antibody interactions to detect exosomes by utilizing both primary and secondary antibodies. Specifically, antibodies targeting tetraspanin proteins such as CD9 and CD63, which are well-known surface markers of exosomes, are used for detection [15,16]. ELISA offers high sensitivity and allows for the simultaneous analysis of multiple samples. However, it also has limitations: it requires labeling agents for signal detection, involves a complex and time-consuming procedure, and depends on the availability of kits for specific antigen–antibody reactions, such as those targeting CD9 and CD63. Consequently, the method may be less effective for the specific analysis of EVs with complex surface characteristics originating from different sources, as it relies on pre-established antigen–antibody interactions [17,18,19,20,21].

Other methods for EVs’ analysis include nanoparticle tracking analysis (NTA) and flow cytometry. NTA tracks the Brownian motion of particles in a solution using laser illumination, detecting light scattered by the particles to measure the number of exosomes present in the sample [22,23,24,25,26]. This technique offers fast analysis and real-time measurements but has limitations when other particles of similar size are present, making it difficult to selectively analyze exosomes [27]. Flow cytometry, on the other hand, uses fluorescent markers to simultaneously detect the relative size and surface markers of particles, allowing for analysis of EVs [28,29,30]. However, it requires fluorescent-labeled antibodies, and the sample preparation and separation process can be complex, leading to potential sample damage. Moreover, interference between fluorescent signals can limit the method’s sensitivity for quantitative analysis.

To overcome the limitations of traditional analysis methods, recent studies have employed surface plasmon resonance (SPR), an optical technique that measures changes in the refractive index of light through the resonance of free electrons on the surface of metal sensor chips. SPR has been used for the analysis of exosomes in cancer diagnostics [31,32,33,34]. These studies are based on findings that specific antigen proteins are overexpressed in EVs derived from certain cancer cells. By modifying the sensor chip surface with antibodies that selectively bind to these overexpressed proteins, researchers have been able to analyze exosomes with high specificity. For instance, studies have reported the use of a chip surface modified with a CD63 aptamer for the analysis of breast cancer-derived exosomes [35,36], a chip surface functionalized with anti-CD41b for the detection of liver cancer-derived exosomes [37], and a chip modified with anti-EGFR and anti-PD-L1 for the analysis of lung cancer-derived exosomes [38]. Compared to ELISA, SPR-based methods allow for faster, real-time, label-free analysis and can detect exosome levels that may not be measurable with traditional ELISA techniques.

Based on these findings, this study aims to propose a novel method for the quantitative analysis of EVs from previously unreported origins using SPR measurement techniques. We selected EVs derived from *Lactobacillus paracasei* (Lp-EVs) as the target EVs, given recent reports highlighting their anti-inflammatory and anti-aging effects [39,40], suggesting their potential as active substances for therapeutic and cosmetic applications. Developing a quantitative analysis method for these EVs is thus highly relevant. To analyze Lp-EVs using SPR, it is necessary to develop a sensor chip surface that incorporates receptors capable of selectively detecting these EVs. However, little is known about the proteins overexpressed on the surface of these EVs or their surface characteristics. Therefore, we hypothesized that these EVs might exhibit surface protein expression patterns similar to other EVs derived from the same genus. To test this hypothesis, we analyzed the presence of known surface protein markers.

Among the proteins commonly associated with *Lactobacillus*-derived EVs, p40 and p75 have attracted considerable attention due to their reported biological activities. These proteins, originally characterized in *Lactobacillus rhamnosus* GG and *Lactobacillus casei*, are known to exert anti-inflammatory and anti-apoptotic effects, particularly by modulating host epithelial responses [41,42,43,44]. p40 has been shown to activate the epidermal growth factor receptor (EGFR) pathway, thereby promoting cell survival and barrier protection, while p75 has been linked to mucosal immune modulation and inhibition of apoptosis. These functional properties suggest that EVs containing p40 and p75 may serve as potent therapeutic agents in both intestinal and skin-related applications.

Through this process, we identified specific proteins that are overexpressed on the surface of Lp-EVs and developed a sensor chip surface capable of selectively detecting these exosomes. This proposed method enables the analysis of overexpressed surface features of novel EV types, facilitating the development of sensor chips for specific detection and quantitative analysis of the target EVs. Additionally, this approach could be effectively used to determine appropriate dosages of exosomes as therapeutic agents, ensuring proper formulation management for products containing these exosomes.

## 2. Materials and Methods

### 2.1. Materials

*Lactobacillus paracasei*-derived EVs (Lp-EVs), *Bacillus amyloliquefaciens*-derived EVs (Ba-EVs), and *Enterococcus rotai*-derived EVs (Er-EVs) were provided by CHA Meditech Co., Ltd. (Pocheon, Republic of Korea). Recombinant anti-p40 ΔNp63 antibody, Alexa Fluor^®^ 647 anti-p40 ΔNp63 antibody, and FITC anti-p75 NGF receptor antibody were obtained from Abcam (Cambridge, UK). Strat-M^®^ membranes were purchased from Merck Millipore (Seoul, Republic of Korea). N-(3-Dimethylaminopropyl)-N′-ethylcarbodiimide hydrochloride (EDC), N-Hydroxysuccinimide (NHS), ethanolamine hydrochloride, and the PKH67 Green Fluorescent Cell Linker Mini Kit for General Cell Membrane Labeling were purchased from Sigma-Aldrich Korea Ltd. (Seoul, Republic of Korea) pH 4.0 acetate buffer was obtained from ICLUEBIO Co., Ltd. (Ansan, Republic of Korea).

### 2.2. Preparation of Lp-EVs

The *Lactobacillus paracasei* strain used for EV production was previously isolated from the human body and genetically characterized by MD Healthcare Inc. (Seoul, Republic of Korea) [39]. Lp-EVs were prepared as previously described [39]. Briefly, *Lactobacillus paracasei* was inoculated into a proprietary EMP medium developed by MD Healthcare and cultured at 37 °C with shaking at 200 rpm until the optical density at 600 nm reached 1.0–1.5. The culture was harvested and centrifuged at 10,000× *g* for 20 min at 4 °C to remove cells. The supernatant was filtered through a 0.22 μm bottle-top filter (Corning, Corning, NY, USA) and concentrated 200-fold using a MasterFlex peristaltic pump system (Cole-Parmer, Vernon Hills, IL, USA) equipped with a 100 kDa Pellicon 2 Cassette filter (Merck Millipore, Burlington, MA, USA). The concentrate was filtered again through a 0.22 μm membrane and subjected to ultracentrifugation at 150,000× *g* for 3 h at 4 °C. The final Lp-EV pellet was resuspended in PBS and stored at −80 °C. The protein concentration of Lp-EVs was determined using a bicinchoninic acid (BCA) protein assay kit (Thermo Fisher Scientific, Waltham, MA, USA). This protein-based quantification approach followed the procedure described in [40], which we previously used to characterize Lp-EVs. To ensure batch-to-batch consistency, we standardized the EV isolation, concentration, and quantification protocols. Only EV preparations that exhibited comparable total protein content and particle size distributions, determined by nanoparticle tracking analysis (NTA), were used for further experiments. Additionally, the reproducibility of biological activity was confirmed across three independently prepared EV batches, ensuring the reliability of downstream functional analyses.

### 2.3. Characterization of Exosomes

#### 2.3.1. Transmission Electron Microscopy (TEM)

The morphology of the exosomes was observed using an energy-filtering transmission electron microscope (LIBRA 120, Carl Zeiss, Jena, Germany). A 5 µL aliquot of the exosome solution was applied to a carbon-coated grid and allowed to adsorb for 1 min, after which excess solution was blotted off using filter paper. For staining, 100 µL of 2% uranyl acetate was placed on a piece of parafilm, and the grid, with the exosome-adsorbed side facing down, was placed onto the droplet for 10 s. Excess uranyl acetate was then blotted off with filter paper. Finally, the grid was thoroughly dried before TEM imaging.

#### 2.3.2. Dynamic Light Scattering Analysis and Zeta Potential Measurements

The size distribution and zeta potential of the exosomes were measured using an ELSZ-2000ZS analyzer (Otsuka Electronics, Osaka, Japan). The exosome solution was diluted in PBS and filtered through a 0.22 µm filter. A 2 mL aliquot of the filtered exosome solution was transferred into a cuvette, which was then placed in the DLS instrument to measure the size distribution. Additionally, a 1 mL aliquot of the same sample was used to measure the zeta potential.

#### 2.3.3. Flow Cytometry Analysis

Specific binding interactions between exosomes and antibodies were analyzed using a Cytoflex flow cytometer (Beckman Coulter, Brea, CA, USA). Flow cytometry was performed using a CytoFLEX V4-B5-R3 Flow Cytometer (Product No: B53001, Beckman Coulter, Brea, CA, USA), which allows direct detection of nanoparticles down to <100 nm in diameter using its violet side scatter (VSSC) detection system. EV samples were diluted in PBS, with 200 µL prepared for each sample. The samples were then labeled with Alexa Fluor^®^ 647 anti-p40 ΔNp63 antibody by adding 2 µL of the fluorescent antibody to each sample, followed by incubation at 4 °C for 30 min. During incubation, the samples were gently pipetted every 15 min to ensure thorough mixing and labeling. To verify antibody labeling on the exosomes, the *x*-axis of the analysis graph was set to detect the emission wavelength of Alexa Fluor^®^ 647 at 660/20 BP. Unlabeled exosomes were used as the control. The labeled exosome samples were then analyzed, and changes in the *x*-axis signal compared to the control indicated the interaction between the three types of exosomes and the antibody.

#### 2.3.4. Western Blot Analysis

A Western blot analysis was conducted to examine the specific interactions between the three types of EVs and the antibody. Each exosome sample (20 µg) was dissolved in buffer containing 2-mercaptoethanol, boiled for 5 min, and then subjected to protein separation using a 10% sodium dodecyl sulfate-polyacrylamide (SDS-PAGE) gel. The separated proteins were transferred onto a PVDF membrane, which was then blocked with 5% skim milk for 1 h. The membrane was incubated overnight at 4 °C with anti-p40 ΔNp63 antibody diluted 1:1000 in 5% skim milk. Following this, the membrane was treated with anti-rabbit IgG and HRP-linked antibody, diluted 1:5000 in 5% skim milk, for 1 h at room temperature. The final detection was performed using the Amersham Imager 680 system (General Electric, Boston, MA, USA), and the results were analyzed using the same imaging system.

### 2.4. SPR Measurements for Lp-EVs Detection

SPR analysis was conducted using the iMSPR-mini system (ICLUEBIO, Co., Ltd.). A gold chip functionalized with 11-mercaptoundecanoic acid (11-MUA) was immobilized onto an SPR prism using matching oil. A mixed solution of 0.4 M EDC and 0.1 M NHS in PBS was flowed over the gold chip for 5 min to activate the carboxyl groups (–COOH). Anti-p40 ΔNp63 antibody was diluted in pH 4.0 acetate buffer to a concentration of 10 µg/mL. This antibody solution was then introduced to the SPR device and flowed over the activated gold chip surface for 5 min to facilitate the EDC/NHS reaction. Subsequently, 1 M ethanolamine was flowed over the chip for 5 min to block any nonspecific binding sites that had not reacted with the antibody, resulting in a gold chip with immobilized antibody. The fabricated chip was then exposed to three types of EVs at varying concentrations to detect the interaction signals between the antibodies and the EVs. Throughout the entire process, the flow rate was consistently maintained at 30 µL/min. PBS was used for washing between each step of the experiment. The SPR measurements were performed using a dual-channel system, in which one channel was immobilized with anti-p40 antibody, while the other channel was treated with PBS as a reference control. During analysis, the instrument automatically subtracted the reference signal from the experimental signal to eliminate non-specific binding and baseline drift. All experiments were conducted in triplicate.

### 2.5. Analysis of Lp-EVs on the Skin Membrane Surface Using SPR Measurements

The analysis of the EVs on an artificial skin membrane was conducted using the FDC-6T system (LOGAN, Somerset, NJ, USA), utilizing the Strat-M^®^ Membrane (Merck Millipore, USA) as the skin membrane. The membrane was positioned in a Franz diffusion cell with the surface facing upward. To evaluate EV concentration at the skin membrane surface, experiments were performed at six time points: 0, 1, 3, 6, 15, and 24 h. A 0.5 mL aliquot of EVs, diluted to 250 µg/mL in PBS, was applied to the membrane surface, while the receptor chamber was filled with PBS and stirred at 600 rpm at 37 °C. At each designated time point, a small volume of the EVs solution was retrieved from the membrane surface and diluted in PBS. The collected samples were quantitatively analyzed using SPR to assess changes in EV concentration. All experiments were performed in triplicate.

### 2.6. Fluorescence Analysis of Lp-EVs on the Skin Membrane Surface

#### 2.6.1. Fluorescence Labeling of Exosomes

To visualize the Lp-EVs on the artificial skin membrane using fluorescence imaging, the exosomes were labeled with a fluorescent dye. Labeling was performed using the PKH67 Green Fluorescent Cell Linker Mini Kit. The exosomes were diluted in Diluent C to a concentration of 250 µg/mL, and 6 µL of the dye was added. The mixture was gently pipetted for 30 s and then incubated at room temperature for 5 min. To halt the labeling reaction, 2 mL of 10% BSA was added, and the mixture was centrifuged at 180,000× *g* for 2 h at 4 °C. The resulting EVs pellet was resuspended in 1 mL of PBS and transferred to an Amicon 10 kDa MWCO filter, where it was centrifuged at 2800× *g* for 40 min at 4 °C. After another resuspension in 1 mL of PBS, the final fluorescently labeled exosome sample was prepared for imaging.

#### 2.6.2. Fluorescence Imaging Analysis of Skin Membrane Cross-Section

The Lp-EV samples labeled with the fluorescent dye PKH67 were utilized in the artificial skin penetration experiments. The experimental procedure was conducted as described in Section 2.4. To analyze the cross-sections of the artificial skin at various time points, one sample was prepared for each designated time point. After all artificial skin samples were collected, the surface areas where Lp-EVs had been applied were washed three times with PBS. The samples were then frozen at −80 °C using OCT compound (FSC 22 Clear, Leica, Wetzlar, Germany). The artificial skin was cut into 20 µm thick sections using a CM3050S Cryostat Microtome (Leica, Wetzlar, Germany) to visualize the cross-sections, and the images of the skin cross-sections at different penetration time points were captured using confocal microscopy.

## 3. Results and Discussion

### 3.1. Characterization of Lp-EVs for Identification of Specific Receptors

Due to the complexity and diversity of proteins present on the surface of EVs, we aimed to identify and analyze proteins that are specifically overexpressed on the surface of Lp-EVs. Since EVs tend to express similar or identical proteins depending on their origin, we hypothesized that EVs derived from the same genus, *Lactobacillus*, would share common antigenic proteins. Based on this hypothesis, we limited the scope of surface protein identification. Although surface protein identification for *Lactobacillus paracasei* and its derived EVs (Lp-EVs) has not yet been reported, prior studies have demonstrated that the p40 and p75 proteins are expressed in other *Lactobacillus* species, such as *Lactobacillus rhamnosus* GG and *Lactobacillus casei* [41,45,46,47]. These findings are also supported by previous proteomic analyses of *Lactobacillus*-derived extracellular vesicles [48]. In a study, microvesicles (MVs) secreted by *Lactobacillus casei* BL23 were isolated and subjected to LC-MS/MS-based proteomic profiling. Among the 103 identified proteins, both p40 and p75 were confirmed to be present in the vesicular fraction, suggesting that these proteins are actively secreted in EVs and may contribute to the strain’s probiotic activity. This supports the biological plausibility of our detection system and strengthens the hypothesis that p40 and p75 are conserved EV-associated proteins within the *Lactobacillus casei*/*paracasei*/*rhamnosus* group. Therefore, we conducted a surface characterization analysis under the hypothesis that the p40 or p75 proteins might also be uniquely expressed on Lp-EVs.

**Figure 1:**
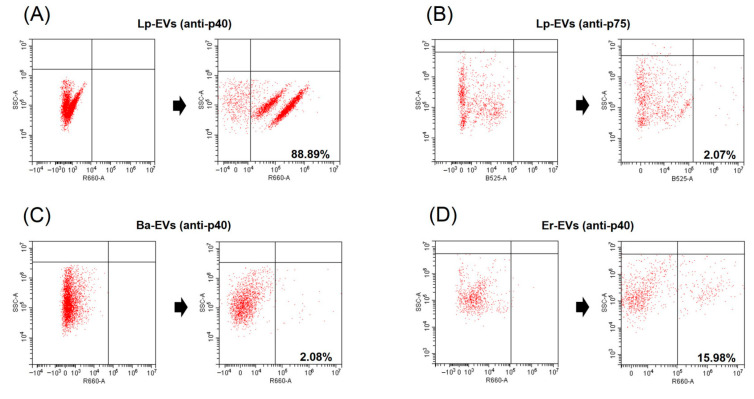
Detection of p40 and p75 proteins from Lp-EVs and p40 protein from Ba-EVs and Er-EVs using FACS. FACS data demonstrate that anti-p40 specifically interacts with Lp-EVs (**A**), with a staining ratio of 88.89%, but shows minimal interaction with Ba-EVs (**C**, 2.08%) and Er-EVs (**D**, 15.98%). Additionally, anti-p75 does not significantly interact with Lp-EVs (**B**, 2.07%). These results indicate the specificity of anti-p40 for Lp-EVs.

We conducted FACS analysis on Lp-EVs using anti-p40 and anti-p75 to verify our hypothesis. The FACS analysis results showed that Lp-EVs exhibited 88.89% specific binding to anti-p40 (Figure 1A), but only 2.08% binding to anti-p75 (Figure 1B). For the control groups, *Bacillus amyloliquefaciens*-derived EVs (Ba-EVs) and *Enterococcus rotai*-derived EVs (Er-EVs) were included and analyzed under the same conditions, as these exosomes originate from sources outside the *Lactobacillus* genus; thus, p40 was not expected to be highly expressed. As expected, Ba-EVs and Er-EVs showed significantly lower binding, with 2.03% (Figure 1C) and 15.98% (Figure 1D), respectively. This indicates that the interaction with anti-p40 was significantly lower in Ba-EVs and Er-EVs compared to Lp-EVs. The results confirm that p40 is relatively overexpressed in Lp-EVs compared to EVs from other genera. Therefore, anti-p40 can be considered a valid receptor for the specific detection of Lp-EVs.

**Figure 2:**
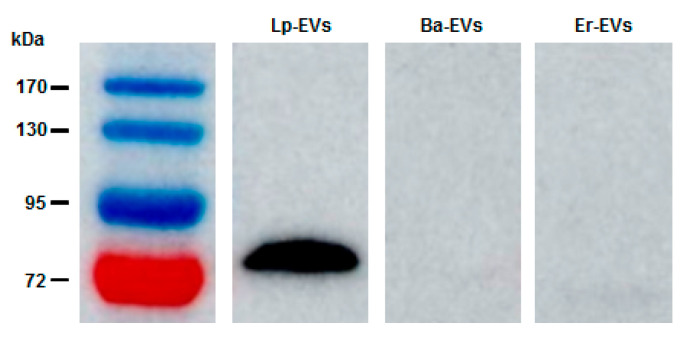
Western blot analysis confirming the FACS results. The data demonstrate that anti-p40 specifically interacts with Lp-EVs, while no interaction is observed with Ba-EVs or Er-EVs.

Western blot analysis was also conducted to examine the interaction between anti-p40 and the three types of EVs: Lp-EVs, Ba-EVs, and Er-EVs. Consistent with the FACS results, anti-p40 interacted with Lp-EVs, producing a distinct band at 75 kDa (Figure 2). In contrast, no bands were observed in the corresponding region for Ba-EVs and Er-EVs, indicating minimal interaction with anti-p40. The detection of p40 expression in Lp-EVs is particularly meaningful in light of its documented biological functions. As previously reported, p40 and p75 contribute to epithelial cell protection by activating EGFR signaling, suppressing pro-inflammatory cytokines, and reducing apoptosis [41,42,43,44]. These effects have been demonstrated in both intestinal and skin epithelial models, highlighting the therapeutic relevance of EVs that express these proteins. Therefore, identifying the presence of p40 on the surface of Lp-EVs not only serves as a useful detection target but also supports the potential applicability of these EVs in treating inflammatory and degenerative skin conditions. These results further confirm that the p40 protein is specifically expressed on the surface of Lp-EVs. Consequently, functionalizing the surface of an SPR sensor chip with anti-p40 could facilitate the specific detection of Lp-EVs through SPR biosensing.

A distinct protein band at approximately 75 kDa was consistently observed in Western blot analysis of Lp-EVs using the anti-p40 antibody. Although this differs from the theoretical molecular mass of p40 (~40 kDa), similar anomalous migration behavior has been reported in previous studies. In particular, Bäuerl et al. demonstrated that p40 proteins in Lactobacillus casei may exhibit reduced electrophoretic mobility, likely due to their highly polar and structurally flexible N-terminal domain, resulting in a higher apparent molecular weight [46]. Moreover, the functional data obtained from flow cytometry and SPR assays further support the selective recognition of this protein in Lp-EVs. Nonetheless, future studies incorporating proteomic validation such as mass spectrometry will be important to definitively confirm the molecular identity of the detected band.

**Figure 3:**
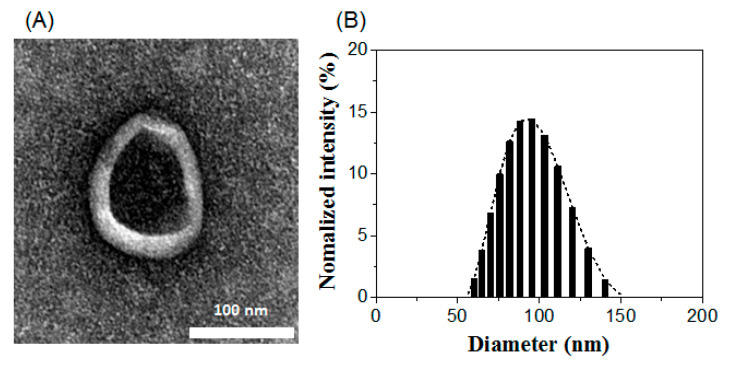
Characterization of Lp-EVs using (**A**) TEM imaging and (**B**) DLS analysis.

Additionally, the physical characteristics of Lp-EVs were analyzed using transmission electron microscopy (TEM) and dynamic light scattering (DLS). As shown in Figure 3A, TEM revealed the presence of a double membrane structure, resulting from staining, and a spherical morphology of the EVs. The diameter of the exosomes was approximately 100 nm. DLS analysis further confirmed the size distribution of the EVs, measuring an average diameter of 93.8 ± 18 nm (Figure 3B), which is consistent with the size observed in the TEM images. The zeta potential of the exosome surface was measured at −17.30 mV, indicating that Lp-EVs carry a negative charge. These findings confirm the morphology, size distribution, and charge of Lp-EVs, which are consistent with the typical characteristics of EVs as reported in previous studies [49,50].

Although a 15.98% binding signal was observed between anti-p40 and Er-EVs, this response was substantially lower than the 88.89% observed with Lp-EVs, highlighting the selective affinity of anti-p40 toward Lp-EVs. In this context, Er-EVs, originating from a different genus, were included as a comparative reference to demonstrate binding specificity rather than to perform an extensive cross-reactivity assessment. The primary focus of this study was to identify a surface marker enriched in Lp-EVs and to validate its applicability for selective detection using an SPR-based platform. Broader evaluations involving EVs from other *Lactobacillus* species or detailed analyses of potential cross-reactivity with non-target genera fall beyond the scope of the current study and are planned as part of our future research.

### 3.2. Specific Detection of Lp-EVs Using Anti-p40-Modified Au Thin Film

**Figure 4:**
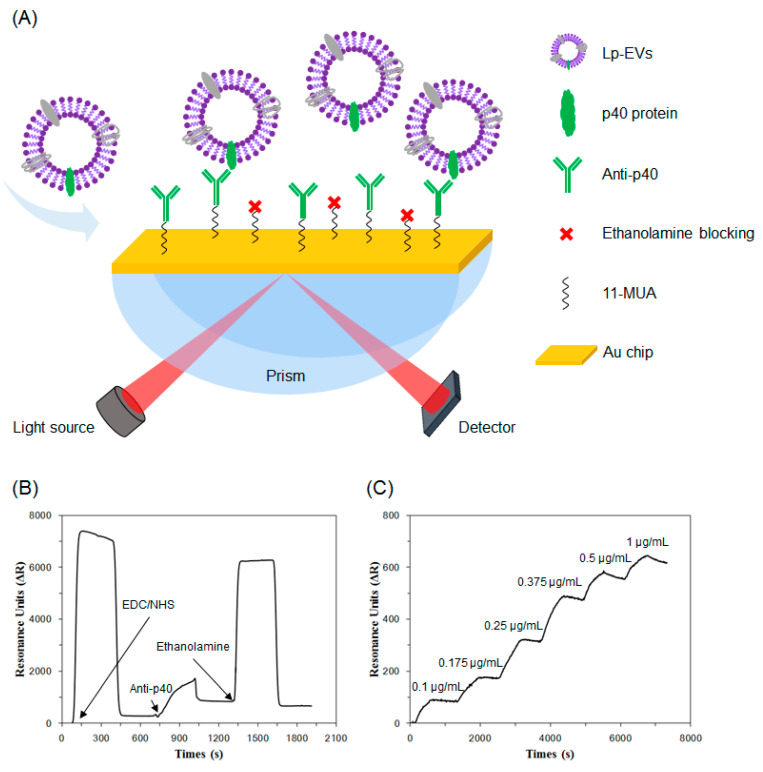
(**A**) Illustration of Lp-EVs expressing p40 protein and the specific detection of Lp-EVs using an anti-p40 modified SPR sensor chip. The graph shows sensor chip surface modification and Lp-EVs detection for quantitative analysis. (**B**) Immobilization of anti-p40 on the sensor chip. (**C**) SPR signal change graph corresponding to Lp-EVs concentration using the fabricated sensor chip.

Based on the surface characterization of Lp-EVs, we fabricated an SPR sensor chip with anti-p40 and measured SPR signal changes in response to varying concentrations of Lp-EVs (Figure 4A). Figure 4B illustrates the time-dependent SPR signal shifts (ΔR) during the antibody immobilization process on the sensor chip, as shown in Figure 4A. To immobilize anti-p40 on the chip, a solution of EDC/NHS was applied, resulting in an SPR signal increase of 282.8 RU. Subsequently, the introduction of 10 µg/mL anti-p40 further elevated the SPR signal by 666.29 RU, indicating successful antibody immobilization. To minimize nonspecific binding, 1M ethanolamine was used to block any remaining unreacted sites on the chip surface.

Using the fabricated sensor chip, we conducted detection experiments with varying concentrations of Lp-EVs. Standard solutions of Lp-EVs at concentrations of 0.1 µg/mL, 0.175 µg/mL, 0.25 µg/mL, 0.375 µg/mL, 0.5 µg/mL, and 1 µg/mL were prepared and introduced to the chip surface in increasing order, while measuring the SPR signal changes over time (Figure 4C). The SPR signal values for each concentration increased accordingly, with values of 71.53 ± 17.24 RU, 195.82 ± 27.03 RU, 408.19 ± 83.60 RU, 621.71 ± 129.25 RU, 704.87 ± 129.76 RU, and 789.97 ± 127.81 RU, respectively (the experiment was repeated three times). The signal reached saturation within approximately 500 s after the introduction of Lp-EVs, indicating that the reaction time between Lp-EVs and the anti-p40 immobilized on the sensor chip surface was around 500 s. These results confirm that as the concentration of Lp-EVs increases, a greater number of exosomes bind to the antibodies on the sensor chip surface, leading to a corresponding increase in the SPR signal.

**Figure 5:**
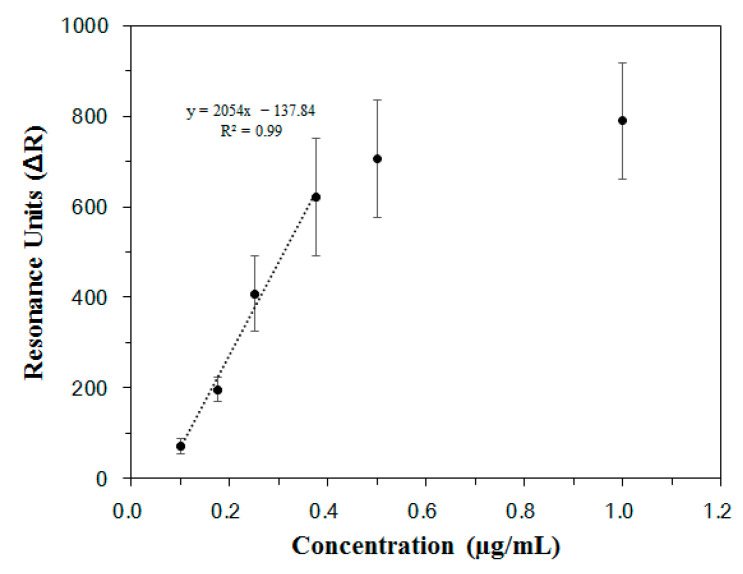
Calibration curve of Lp-EVs by concentration based on SPR signal.

The SPR signal changes measured for each concentration of Lp-EVs in Figure 4C were used to generate a calibration curve. As shown in Figure 5, a linear range was observed between Lp-EV concentrations of 0.1 µg/mL and 0.375 µg/mL, indicating that within this range, unknown concentrations of Lp-EVs could be accurately quantified. The equation for the linear portion of the graph in this concentration range is y = 2054x − 137.84, with a correlation coefficient R^2^ of 0.99, indicating a high degree of linearity and reliability for quantitative analysis. The limit of detection (LOD) for Lp-EVs using the anti-p40 modified SPR sensor chip was determined to be 0.12 µg/mL. Beyond an Lp-EVs concentration of 0.5 µg/mL, the SPR signal reached a plateau, suggesting that the sensor surface became saturated with Lp-EVs at concentrations above 0.5 µg/mL.

To validate the specificity of the anti-p40 modified SPR sensor chip for detecting Lp-EVs, SPR analysis was conducted under the same conditions using Ba-EVs and Er-EVs on the same chip surface. Although the results are not displayed, no significant SPR signal changes were observed. This indicates that the sensing technique utilizing the anti-p40 modified SPR sensor chip is capable of specific detection and quantitative analysis of Lp-EVs, based on the established calibration curve.

Recent advances in SPR-based biosensor technologies have demonstrated the feasibility of detecting extracellular vesicles with high sensitivity and specificity in a label-free manner [51]. Building upon these developments, the present study introduces a unique application of SPR for the quantitative detection of Lp-EVs, which has not been previously explored. By employing an anti-p40-functionalized sensor surface, our platform enables strain-specific EV recognition, distinguishing it from conventional EV assays targeting broadly expressed markers. While there is room for further sensitivity enhancement, the system already offers robust specificity and a promising foundation for microbiome-targeted diagnostic and therapeutic applications.

While the proposed SPR-based detection system demonstrated high specificity and adequate sensitivity for Lp-EVs under controlled conditions, there is room for further improvement. Enhancing detection sensitivity through signal amplification strategies or surface nanostructuring could broaden its applicability to lower-concentration samples. Moreover, the current validation was conducted using purified EV samples; future studies will be required to evaluate the robustness and accuracy of this approach in complex biological matrices such as serum or tissue extracts. Addressing these aspects will help advance the method toward practical biomedical and cosmetic use.

### 3.3. Evaluation of Quantitative Analysis of Lp-EVs on the Skin Membrane

An experiment was designed to validate the potential for quantitative analysis of Lp-EVs using the anti-p40 modified SPR sensor chip and the calibration curve obtained from SPR sensing. Based on previous studies indicating that Lp-EVs are involved in anti-inflammatory and anti-aging mechanisms, in vivo quantitative analysis of Lp-EVs is essential for their use as an active ingredient in pharmaceutical or cosmetic applications. To test this, skin tissue was selected as the target. An artificial skin membrane model was employed, and the Lp-EVs solution was applied to assess the uptake or penetration of Lp-EVs into the skin membrane, which was quantitatively analyzed.

**Figure 6:**
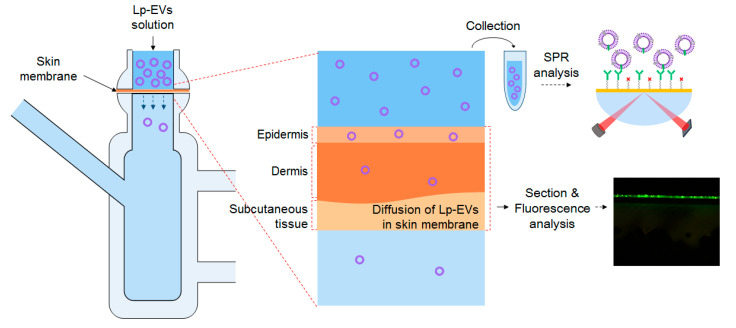
Schematic representation of SPR measurements and fluorescence analysis to evaluate the behavior of sampled Lp-EVs across the skin membrane for Lp-EVs concentration change analysis.

Figure 6 illustrates the schematic diagram for analyzing the diffusion behavior of Lp-EVs across a skin membrane over time, using a Franz diffusion cell. The skin membrane was fixed to the Franz cell, and an Lp-EVs solution was applied to its surface. To quantitatively assess the uptake or penetration of Lp-EVs into the skin membrane, small aliquots of the Lp-EVs solution from the upper chamber of the Franz cell were collected at specific time intervals, diluted, and then introduced to the surface of an SPR sensor chip modified with anti-p40 antibodies. The changes in SPR signal were measured (the experiment was conducted in triplicate).

The concentration of 250 µg/mL used in the topical application of Lp-EVs was selected based on prior studies that optimized this value for fluorescence labeling and skin-related functional analysis. Lower concentrations have been reported to yield insufficient fluorescence signal, while excessively high concentrations can cause signal saturation or distortion [40]. Therefore, the concentration was chosen to ensure consistent visualization and comparability with previous findings.

It should be noted that the linear dynamic range of the SPR calibration curve, as shown in Figure 5, spans from 0.1 to 0.375 µg/mL. To accommodate this, the collected samples from the Franz diffusion cell were appropriately diluted prior to SPR measurement. Although the applied dose was relatively high, the dilution step allowed accurate quantification within the validated linear range. This approach ensures that the SPR platform can be reliably used for both low- and high-concentration samples through pre-measurement adjustment, maintaining the integrity of the quantitative analysis.

The SPR signal values from the collected solutions at 0 h, 1 h, 3 h, 6 h, 15 h, and 24 h were 863.00 (±24.82) RU, 849.73 (±43.18) RU, 814.70 (±31.89) RU, 764.59 (±19.72) RU, 655.68 (±33.92) RU, and 493.54 (±74.70) RU, respectively. By applying these SPR signal values to the calibration curve in Figure 5, the concentrations of Lp-EVs in the collected solutions were quantitatively determined. The initial Lp-EVs concentration of 250 µg/mL (0 h) decreased over time to 240.40 µg/mL (1 h), 231.87 µg/mL (3 h), 218.95 µg/mL (6 h), 193.16 µg/mL (15 h), and 153.70 µg/mL (24 h).

**Figure 7:**
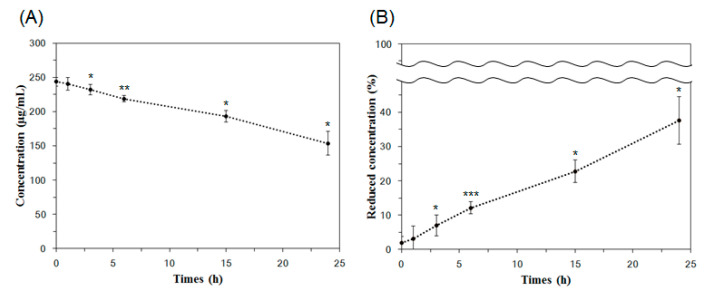
Analysis of Lp-EVs concentration changes over time on the skin membrane surface using SPR measurements. (**A**) Lp-EVs concentrations measured at 1 h, 3 h, and 6 h were 240.40 µg/mL, 231.87 µg/mL, and 218.95 µg/mL, respectively, with significant decreases observed at 15 h (193.16 µg/mL) and 24 h (153.70 µg/mL) from an initial concentration of 250 µg/mL. (**B**) The concentration reduction is expressed as a percentage, showing values of 3.07%, 7.00%, 12.12%, 22.73%, and 37.62% from 1 h to 24 h, respectively. Statistical significance was determined using unpaired two-tailed *t*-tests comparing each time point to the initial concentration (0 h, 250 µg/mL). Asterisks indicate significant differences (* *p* < 0.05, ** *p* < 0.01, *** *p* < 0.001). Error bars represent the standard deviation from triplicate measurements (*n* = 3).

The time-dependent changes in Lp-EVs concentration, as analyzed, are presented in Figure 7A. The decreasing trend in Lp-EVs concentration over time suggests that Lp-EVs gradually diffuse into the skin membrane. A more pronounced decrease in concentration is observed after 15 h, indicating an increased diffusion of Lp-EVs into the membrane during this period. Figure 7B shows a graph depicting the percentage of the initial Lp-EVs concentration remaining at each time point. This reflects the proportion of Lp-EVs that diffused into the skin membrane, with values of 3.07% (1 h), 7.00% (3 h), 12.12% (6 h), 22.73% (15 h), and 37.62% (24 h) relative to the initial concentration. These results suggest that Lp-EVs diffuse into the skin membrane at a relatively consistent rate over time. To further investigate whether the diffusion behavior of Lp-EVs in the skin membrane was due to surface uptake or penetration, a similar analysis was conducted on the solution from the lower chamber of the Franz cell. The SPR signal changes in the lower solution were below the limit of detection (LOD) throughout the time course, indicating negligible Lp-EVs penetration through the skin membrane. Therefore, it can be expected that the diffusion of Lp-EVs is primarily due to uptake into the skin membrane, with minimal penetration through it.

It should be noted that the Strat-M^®^ membrane, while structurally designed to simulate the lipid composition and barrier properties of the human epidermis, lacks dynamic biological processes such as epidermal renewal and appendageal structures (e.g., hair follicles, sweat glands), which may influence percutaneous absorption in vivo. Despite this limitation, the Strat-M^®^ model was adopted in this study due to its reproducibility and practical suitability for initial screening of diffusion behaviors under controlled conditions using SPR measurements. Our primary aim was to demonstrate the feasibility of specific detection and quantitative analysis of Lp-EVs using the developed SPR platform in a simplified and standardized skin-like barrier system. In addition, the biocompatibility of Lp-EVs has been previously evaluated in vitro using human dermal fibroblasts (HDFs) under serum-free conditions [40]. The results showed no significant cytotoxicity at most tested concentrations, except at 100 µg/mL, confirming their general safety at physiologically relevant levels. Notably, Lp-EVs also improved HDF viability under TNF-α-induced inflammatory conditions, suggesting potential therapeutic benefit. These findings support the applicability of Lp-EVs for further skin-targeted studies.

To assess the statistical significance of the concentration changes observed over time, unpaired two-tailed *t*-tests were performed comparing each time point to the initial concentration (250 µg/mL at 0 h). In the absolute concentration data (Figure 7A), the changes at 1 h (*p* = 0.37883) were not statistically significant, whereas significant reductions were observed at 3 h (*p* = 0.03585), 6 h (*p* = 0.00283), 15 h (*p* = 0.02221), and 24 h (*p* = 0.01865). Similarly, in the normalized percentage data (Figure 7B), no significance was observed at 1 h (*p* = 0.41235), but significant decreases were detected at 3 h (*p* = 0.02207), 6 h (*p* = 0.0003), 15 h (*p* = 0.01905), and 24 h (*p* = 0.01882). These results suggest that the accumulation of Lp-EVs within the membrane becomes statistically meaningful starting from 3 h post-application.

To ensure that the observed reduction in EV concentration was not due to degradation over time, we conducted a control experiment in the absence of the membrane. Lp-EVs were incubated under identical conditions for 24 h, and the particle size distribution was measured using DLS at 0, 15, and 24 h. The size profiles remained stable, indicating that the vesicles retained their structural integrity over time and that no significant degradation occurred in the absence of the membrane.

### 3.4. Observation of Lp-EVs in the Skin Membrane Using Fluorescence Microscopy

**Figure 8:**
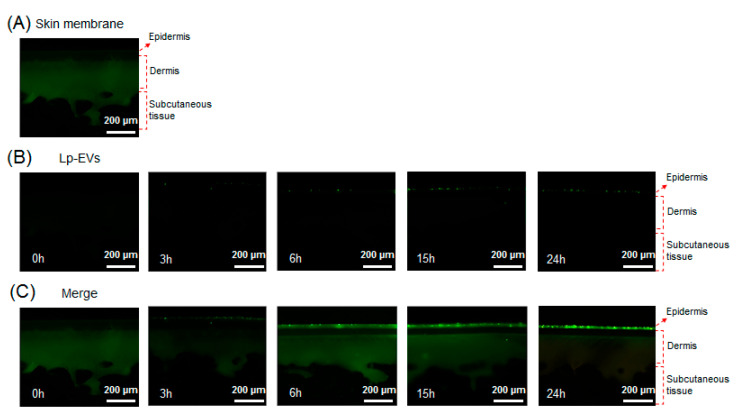
Confocal fluorescence images showing the diffusion of PKH67-labeled Lp-EVs through a Strat-M^®^ membrane model at various time points. (**A**) Native membrane structure visualized via intrinsic autofluorescence, which delineates distinct layers analogous to the epidermis, dermis, and subcutaneous tissue. (**B**) Fluorescence images showing the distribution of Lp-EVs at 0 h, 3 h, 6 h, 15 h, and 24 h. (**C**) Merged images combining Lp-EV signal and membrane autofluorescence to indicate the relative localization of vesicles within membrane layers.

Using confocal microscopy, the behavior of *Lactobacillus paracasei*-derived EVs (Lp-EVs) within a skin membrane was visually analyzed. The membrane exhibits inherent autofluorescence, which enables visualization of distinct membrane layers analogous to the epidermis, dermis, and subcutaneous tissue. Based on these autofluorescence patterns, we labeled each layer in Figure 8 to support the interpretation of Lp-EV diffusion. The merged fluorescence images indicate that Lp-EVs predominantly accumulate in the upper layer, consistent with limited penetration across the membrane barrier over time. Figure 8A shows the fluorescence image of the cross-section of the skin membrane, where the green fluorescence represents the skin membrane’s autofluorescence. Figure 8B displays fluorescence images of Lp-EVs diffused into the skin membrane over a 24-h period, while Figure 8C presents merged images of the original cross-section (Figure 8A) and the fluorescence images of Lp-EVs (Figure 8B). The results indicate that Lp-EVs gradually diffused into the skin membrane over time, with a noticeable increase at 15 and 24 h. This observation is attributed to the fluorescence of PKH67, which was used to label the exosomes, suggesting that the exosomes diffused into the skin membrane. However, fluorescence was observed only in the epidermal layer, indicating that Lp-EVs were confined to the epidermis and did not penetrate the dermal layer. This limited diffusion is likely due to the hydrophobic properties of lipids such as phospholipids, ceramides, and fatty acids in the epidermal and dermal layers, which interfere with interactions between negatively charged Lp-EVs and the skin membrane. Additionally, electrostatic repulsion between negatively charged Lp-EVs and fatty acids may also contribute to this phenomenon [52,53,54]. Despite these limitations, the diffusion of Lp-EVs into the epidermal layer suggests potential for further optimization. Recent studies have explored methods to enhance skin penetration and internal diffusion, including research utilizing microneedle systems to deliver MSC-derived EVs for treating skin aging [55,56] and delivering *Rosa damascena* stem cell-derived EVs for treating melasma [57].

To account for potential background interference due to the autofluorescence of the membrane, both standalone and merged fluorescence images were analyzed at identical membrane cross-sections. Figure 8B displays only the fluorescence signal from PKH67-labeled Lp-EVs, while Figure 8C combines this signal with the membrane’s native autofluorescence. The autofluorescence, primarily observed in the green spectrum, helped delineate the layered structure of the membrane, including the epidermal and dermal analogs, enabling spatial interpretation of Lp-EVs distribution. These annotations help distinguish the PKH67 signal from background autofluorescence and support the conclusion that Lp-EVs were confined to the epidermal layer without penetrating into the dermis. This approach reinforces the qualitative reliability of our fluorescence-based penetration assessment.

A comparative analysis of the fluorescence imaging results from Figure 8 and the quantitative analysis of Lp-EVs diffusion into the skin membrane over time using SPR data from Figure 7 indicates that while the amount of Lp-EVs diffused into the skin membrane increased over 24 h, the exosomes did not completely penetrate the skin membrane but remained confined to the epidermal layer. To further validate this observation, the lower chamber solution of a Franz diffusion cell was sampled at various time points and analyzed quantitatively using an SPR sensor chip. The results revealed that Lp-EVs were barely detectable within the calibration range of the SPR system. This finding supports the interpretation that Lp-EVs primarily accumulate in the epidermal layer without permeating deeper into the dermal layer or passing through the skin membrane.

The restricted diffusion of Lp-EVs to the epidermal region is likely due to their surface physicochemical properties. Zeta potential analysis revealed that Lp-EVs carry a negative surface charge (–17.30 mV), which may lead to electrostatic repulsion when interacting with the similarly negatively charged membrane surface. Supporting this interpretation, a previous study [48] demonstrated that negatively charged liposomes remained on the membrane surface, while positively charged liposomes penetrated deeper into the dermis. These results suggest that the charge-based interactions between Lp-EVs and the membrane, in combination with the hydrophobic nature of the skin lipid matrix, contribute to their epidermal retention.

The findings of this study suggest that Lp-EVs may have beneficial effects on human health by leveraging the known bioactivities of their molecular components. Notably, Lp-EVs express surface proteins such as p40 and p75, which have been reported to exert anti-inflammatory, anti-apoptotic, and epithelial barrier-protective effects in previous studies on *Lactobacillus casei* and *L. rhamnosus* [58,59]. Although our study focused on the detection and permeation characteristics of Lp-EVs, the specific expression of these proteins, combined with the observed retention in the upper skin layer, suggests potential applications in topical delivery systems for modulating skin immunity or improving skin barrier function [60]. These features position Lp-EVs as promising candidates for microbiome-derived therapeutic or cosmetic strategies aimed at enhancing skin health and overall well-being [58].

## 4. Conclusions

This study developed a novel SPR-based method for the specific detection and quantitative analysis of Lp-EVs. Functionalization of a gold thin film with anti-p40 antibodies enabled high specificity and sensitivity, with a detection limit of 0.12 µg/mL. Characterization confirmed the overexpression of p40 protein on Lp-EVs, validating it as a selective detection target. Quantitative analysis of Lp-EVs diffusion into artificial skin membranes revealed their accumulation in the epidermis without dermal penetration, attributed to the lipid-rich and hydrophobic skin barrier and the negatively charged Lp-EVs. Fluorescence imaging confirmed these findings, highlighting their potential for targeted epidermal therapies. The SPR platform offers real-time, label-free, and highly specific detection, making it a valuable tool for advancing research on EV-based therapeutic and cosmetic applications. Future efforts will focus on optimizing delivery systems, such as microneedles, to improve skin penetration and exploring the molecular mechanisms underlying Lp-EVs’ therapeutic effects.

## Data Availability

Data are contained within the article.
